# Transcranial Direct Current Stimulation as an Approach to Mitigate Neurodevelopmental Disorders Affecting Excitation/Inhibition Balance: Focus on Autism Spectrum Disorder, Schizophrenia, and Attention Deficit/Hyperactivity Disorder

**DOI:** 10.3390/jcm11102839

**Published:** 2022-05-18

**Authors:** Beatriz Sousa, João Martins, Miguel Castelo-Branco, Joana Gonçalves

**Affiliations:** 1 Coimbra Institute for Biomedical Imaging and Translational Research (CIBIT), University of Coimbra, 3000-548 Coimbra, Portugal; beatrizmpsousa@gmail.com (B.S.); joao.martins@icnas.uc.pt (J.M.); mcbranco@fmed.uc.pt (M.C.-B.); 2 Institute for Nuclear Sciences Applied to Health (ICNAS), University of Coimbra, 3000-548 Coimbra, Portugal; 3 Faculty of Medicine, University of Coimbra, 3000-548 Coimbra, Portugal

**Keywords:** neurostimulation, neurodevelopmental disorders, GABA, glutamate

## Abstract

Transcranial direct current stimulation (tDCS) has been proposed as a promising therapy for rehabilitation of neurodevelopmental disorders. In this review, we discuss studies on the impact of tDCS as a therapy for autism, schizophrenia, and attention deficit/hyperactivity disorder, as well as the tDCS’ mechanism of action, and propose future paths of research to optimize tDCS treatment protocols. The mechanism underlying tDCS effects is the modulation of excitatory and/or inhibitory activity, making it a valuable tool for restoring the excitation/inhibition (E/I) balance which is disrupted in many neurodevelopmental disorders. Clinical studies have shown that tDCS therapy is well-tolerated by patients and seems to ameliorate behavior and cognitive functions. Alterations in early development of neuronal circuits lead to disruptions in brain activity in neurodevelopmental disorders. An increasing amount of research into the effects of tDCS on neuronal activity has provided a foundation for its use as a therapy for behavior and cognitive characteristics of neurodevelopmental disorders. Clinical studies show that tDCS appears to ameliorate behavioral and cognitive outcomes of patients with autism, schizophrenia, and attention deficit/hyperactivity disorder. More research is needed to understand the mechanisms of action of tDCS and to optimize treatment protocols.

## 1. Introduction

Neurodevelopmental disorders encompass a variety of disorders resulting from abnormal brain maturation and are characterized by a range of deficits in communication, cognition, behavior, and motor skills [1]. Although each disorder is characterized by specific core symptoms, there can be symptom overlap or even co-morbidity [2]. Several studies have suggested imbalance of excitation and inhibition (E/I imbalance) as a common mechanism in a broad set of neurodevelopmental disorders [3,4]. Early development processes, such as formation and migration of neurons, synapse formation and maturation, and refinement of these synapses lead to neural circuits in which excitatory and inhibitory outputs are well balanced [5]. This is important to ensure that response to stimuli is adequate and network activity is well-adjusted and properly timed [4,6]. On the other hand, changes in excitatory transmission are associated with synaptic plasticity—the ability of neural circuits to modify their connections based on changing external outputs—and therefore learning and memory. These changes are also importantly regulated by inhibitory activity [5].

**Figure 1:**
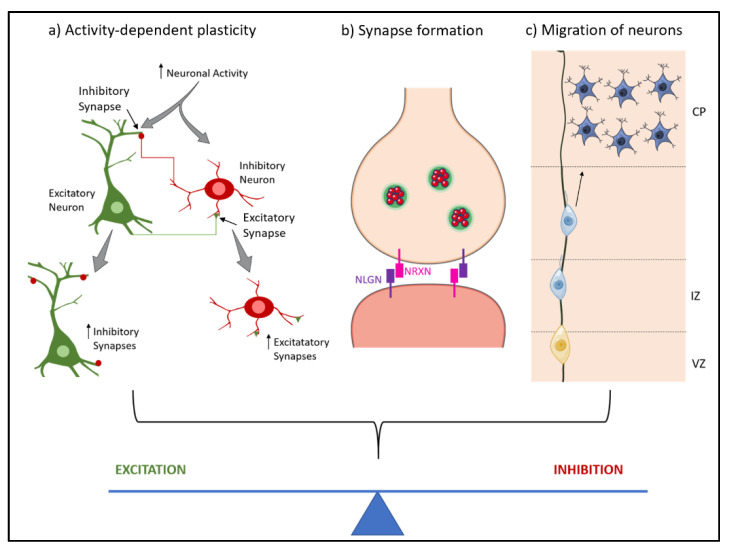
Neurodevelopmental stages that contribute to the excitation/inhibition balance. During the development of the nervous systems, several processes lead to the correct formation and connection of neuronal circuits, achieving an excitation/inhibition balance. These processes are (**a**) Activity-dependent plasticity: neuronal activity induces formation of new synapses; (**b**) Synapse formation: synapses form through cell-cell recognition processes where neurexin and neuroligin form complexes; (**c**) Migration of neurons: the migration of neurons to the appropriate layers is necessary for the correct functioning of neuronal circuits. NRXN—Neurexin; NLGN—Neuroligin; CP—Cortical Plate; IZ—Intermediate Zone; VZ—Ventricular Zone.

Molecules associated with the regulation of E/I balance appear in the early postnatal period. During early postnatal development, changes in number, distribution and type of γ-aminobutyric acid (GABA) and glutamate receptors, as well as the reversal of polarity of GABA from depolarizing to hyperpolarizing, lead to an increase of inhibitory activity and a decrease of excitatory activity, respectively [7]. Further, delay in maturation of inhibitory synapses creates a window for activity-dependent plasticity to occur during early stages of development [8]. The onset of multiple neurodevelopmental disorders matches this critical period [5]. Another important step in the development of neural circuits is the migration of neurons to the correct cortical layer. This allows for different classes of neurons to interact in a balanced way [9]. For instance, E/I balance in cortical layers 2 and 3 appears to play a role in the tuning of neural circuits [10]. In summary, when a shift in the level of either excitation or inhibition occurs, balance is achieved at a different activity level leading to less efficient information processing (Figure 1). This can cause the behavioral symptoms often observed in neurodevelopmental disorders.

This review will focus on the use of transcranial direct current stimulation (tDCS) as a possible therapy for three common neurodevelopmental disorders involving impaired E/I—autism spectrum disorder (ASD), schizophrenia (SCZ), and attention deficit/hyperactivity disorder (ADHD).

## 2. Transcranial Direct Current Stimulation

TDCS is a non-invasive brain stimulation technique that uses constant weak current delivered transcranially—through electrodes placed on the scalp—to modulate neuronal activity [11]. Since weak current is used, tDCS is considered as a neuromodulator technique which does not generate action potentials. Instead, this technique modulates resting membrane potential of neurons, and consequently their probability of firing, altering cortical excitability [12,13].

The efficacy of tDCS is influenced by a variety of factors that should be taken into consideration when applying the technique. These factors can be dependent on the subject, namely genetic polymorphisms, anatomy, and psychological state, which make it harder to predict the effect. However, other factors can be controlled to improve the probability of success. These include the electrode size and positioning, the current intensity and the duration of the stimulation [12]. Typically, two electrodes are used for tDCS, although montages with more than two electrodes are also possible, to focus the stimulation pattern. One electrode is placed on the scalp over the target area, while the other electrode is placed on another area of the scalp or at an extracephalic area. The positioning of the electrodes determines the direction of the current as well as the polarity of the stimulation, which influence the biological effects of tDCS [14]. In cathodal tDCS, the cathode is placed over the area of interest. In this case tDCS decreases cortical excitability by causing hyperpolarization of resting membrane potential that decreases the probability of neural firing, weakening glutamatergic synapses. On the other hand, anodal tDCS, where the anode is placed over the target area, is believed to be mainly excitatory, because it depolarizes the membrane potential, increasing the likelihood of firing, strengthening glutamatergic synapses [12]. Long-term effects of tDCS on plasticity seem to affect the regulation of neurotransmitters, such as dopamine, acetylcholine and serotonin, as well as membrane ion channels [15]. Local changes in ionic concentrations of H^+^ and Ca^2+^ and levels of cyclic adenosine monophosphate (cAMP) have also been described as after-effects of tDCS, along with changes in protein synthesis and modulation of the efficacy of N-methyl-D-aspartate (NMDA) receptors [16].

It has been hypothesized that changes in GABA induced by tDCS are associated with learning and performance improvement. The more GABA is decreased, the larger the learning effect. Disinhibition may allow for activity-dependent long-term potentiation (LTP), which can lead to cortical reorganization. This would explain why tDCS can improve network processing [17].

Since the effects of tDCS can spread through the cortex other than the stimulated area and each one brain area has multiple functions, it is valid to question the spatial specificity of this technique. However, specificity can be achieved with some strategies. Activity-selectivity, for example, is based on the theory that active networks are more sensitive to modulation by tDCS than inactive networks. Therefore, specific activity can be targeted by combining tDCS with training (e.g., learning of motor-skills, cognitive training) [18].

Katz and colleagues [19] have reported significant improvement of working memory (WM) when using a WM task combined with anodal tDCS over the right or left dorsolateral prefrontal cortex (DLPFC), in healthy adults aged between 18 and 35. The authors further reported that WM improvements were more significant compared to sham when baseline ability was lower, suggesting that this approach might be more beneficial for population with low baseline WM abilities. The improvements achieved with this protocol appear to be stable for up to one year. 

**Figure 2:**
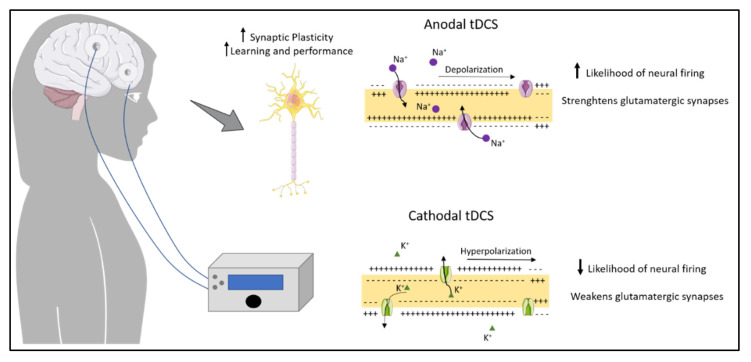
Effects of anodal and cathodal transcranial direct current stimulation (tDCS). The figure represents cortical neurostimulation that could increase synaptic plasticity, leading to improvements in learning and memory. Anodal stimulation increases the likelihood of neuronal firing, strengthening glutamatergic synapses and increasing excitation. Cathodal stimulation decreases likelihood of neuronal firing, weakening glutamatergic synapses and decreasing excitation.

In summary, tDCS modulates network activity by modifying the firing probability of neurons. These effects can be sustained through a series of mechanisms that lead to changes in GABA and glutamate neurotransmitters, cortical reorganization, and increased plasticity (Figure 2). Changes induced by stimulation are associated with learning and improved performance, and specificity can be achieved by combining tDCS with specific tasks.

### 2.1. TDCS as Treatment for Neurodevelopmental Disorders

By facilitating a restoration of balance of neural activity, tDCS may represent a potential therapy for neurodevelopmental disorders in which E/I imbalance plays a central role. In fact, several pre-clinical and clinical studies have been performed, or are underway, on the effects of tDCS in several neurodevelopmental disorders, including ASD, SCZ, and ADHD. In the following sections, we describe multiple studies on animals and humans for the neurodevelopmental disorders this review focuses on. It is worth noting that the majority of these studies reported significant results. However, this may also be a consequence of publication bias, defined as a bias against publishing of non-significant data, or data that goes against a popular hypothesis.

#### 2.1.1. Effect of tDCS on Autistic-like Behavior

According to DSM-5 [20], ASD core symptoms are deficits in social communication and interaction, repetitive behaviors and restricted interests. These cognitive deficits may also be accompanied by intellectual disability. Symptoms may vary in severity from mild, often designated high-functioning ASD—no intellectual or verbal disability and easily masked by coping strategies—to very severe early childhood autism—lack of verbal language and inability to cope independently [21]. Due to the heterogeneity of symptoms, a definitive explanation for the underlying mechanisms has been challenging. 

Some authors have suggested that alterations in the metabolism of GABA and glutamate leads to the E/I imbalance observed in ASD patients, and this is the cause of autistic-like behavior [22,23]. In fact, many of the genes associated with ASD are involved in the development of both excitatory and inhibitory neurons [24], formation and maturation of synapses, and synaptic plasticity and function expression [25]. Moreover, irregularities in cortical column integrity—defined as the most basic functional unit of neural organization—can lead to deficient connectivity between distant cortical regions and impaired GABAergic regulation [26,27]. 

MRS studies performed on both animal models [28] and humans [29,30] have further provided evidence for the altered ratio of GABA and glutamate, and deficient GABA signaling [31], in the autistic brain. If indeed autistic-like behaviors are caused by an E/I imbalance, tDCS could provide a solution for the alleviation of such behaviors. 

Indeed, a study carried out on six adults (18–58 years of age) with high-functioning ASD using anodal tDCS (2.0 mA; 30 min) applied over the right temporoparietal junction (TPJ) to investigate whether it could improve measures of social cognition and social skills reported significant differences between tDCS and sham on verbal fluency tests for emotion categories, concluding that tDCS may indeed improve emotional processing in individuals with ASD [32]. Accordingly, another work performed on minimally verbal children with ASD, also using anodal tDCS (0.08 mA/cm^2^; 30 min) applied to the dorsolateral prefrontal cortex (DLPFC), showed an improvement of syntax acquisition after treatment [33]. Goméz and colleagues [34] also observed a significant reduction in the total score on the Autism Behavior Checklist, Autism Treatment Evaluation Checklist, and the Autism Diagnostic Interview during the first six months after treatment. Children diagnosed with slight and moderate ASD received twenty daily sessions of tDCS applied to the left DPLFC (1 mA, 20 min) and their performance on three clinical scales was evaluated before and one, three, and six months after completing the sessions. The authors reported a significant decrease in the total score of the clinical scales, accompanied by an improvement in autistic behavior one month after the stimulation and the improvements seemed to be maintained until the sixth month after. Electroencephalogram (EEG) functional connectivity analysis showed that brain stimulation also resulted in an increase in brain functional connectivity. Other studies on tDCS for ASD reported improvements in the Autism Treatment Evaluation Checklist (ATEC) social and health and behavioral problems subscale [35], and working memory [36]. Currently, Luckhardt and colleagues [37] are investigating the effects of repeated multi-channel 2 mA anodal stimulation of the bilateral tempo-parietal junction, combined with computer based cognitive training, in children and adolescents with ASD. Also, in teenagers with autism, it was recently reported that multisession tDCS with left DLPFC cathode placement and right supraorbital region anode placement, paired with concurrent cognitive remediation training, promoted social functioning in individuals with ASD [38]. In this study, the authors reported that significantly more participants in the active tDCS group experienced short-term itchiness over the stimulation site than participants in the sham tDCS group. However, other side effects were not significantly different between participants in the active and sham groups [38] Studies are summarized in Table 1.

#### 2.1.2. Effect of tDCS on Schizophrenia

SCZ is a heterogenous disorder that can be differently expressed according to the individual. DSM-5 [20] requires that at least one of the symptoms is delusions, hallucinations, or disorganized speech. Negative symptoms include flattened affect, loss of interest, and emotional withdrawal. Cognitive symptoms present before the onset of psychosis and the severity of these impairments is a predictor of the long-term functional outcome of the disorder [39]. However, these symptoms are also present in other neurodevelopmental disorders, such as ASD and attention deficit/hyperactivity disorder (ADHD), which presents a challenge for early diagnosis [20].

Much like other neuropsychiatric disorders, SCZ appears to be caused by the interaction of environmental factors—e.g., living in an urban environment and drug abuse—and genes [40,41]. Several of the genes identified to increase the risk of developing the disorder are implicated in spine density and morphology (NRG1, DISC1), neurotransmitter metabolism (DAO, DRD4, PPP3CC), serotonin transporter (SLC6A4), and their regulation (COMT, DTNBP1, RGS4) [42]. Spiny synapses are essential units of excitatory neural circuits and necessary for neurotransmitter signal transduction. Therefore, alterations in their morphology or quantity, may lead to changes in excitatory activity [43]. Moreover, an imbalance in neurotransmitters, such as dopamine and GABA, has been suggested to be the reason behind the cognitive deficits and other symptoms of SCZ [39,44]. Alterations in white and grey matter structure have been observed in patients, and diffusion tensor imaging (DTI) studies have shown deficits in white matter integrity. These are signs of alterations in neural connectivity, which has been suggested as the cause for hallucinations [45]. Moreover, MRS studies performed on patients with SCZ have shown altered levels of GABA [46] and glutamate [47], suggesting a possible use for tDCS as a therapy for SCZ.

Hadar and colleagues subjected an adolescent mouse model of SCZ to either anodal or cathodal tDCS (50 µA) to the PFC for 20 min, twice a day. They inferred that anodal tDCS was able to prevent positive neurobehavioral symptoms mimicking SCZ, pointing to a new possible approach in the prevention of the development of the disorder in high-risk individuals [48].

In patients with SCZ, tDCS seems to have a beneficial effect on auditory and verbal hallucinations, as reported by Brunelin et al. [49]. This study followed thirty patients with SCZ, of which fifteen patients received tDCS twice daily for five days, where the anode was placed over the L-DPLFC and the cathode over the left temporoparietal junction (TPJ). Stimulation was set at 2 mA for 20 min. Patients showed a significant improvement on Auditory Hallucination Rating Scale (AHRS) compared to sham, and the effect was maintained for three months. Further, a study with one hundred patients with SCZ (18–25 years of age) where tDCS (2 mA) was administered twice daily for five consecutive days, showed amelioration of negative symptoms, except passive/apathetic withdrawal and stereotyped thinking, that lasted six weeks after the end of the trial [50]. The montage used was the same as used by Brunelin et al. [49]. The study further reported that tDCS did not cause any serious adverse effects. The authors also observed that treatment resistance affected effectiveness of tDCS, decreasing its efficacy [50]. In an interesting study, forty-nine SCZ patients were subjected to tDCS, combined with cognitive training. The subjects performed eight training sessions (two session/day) and received tDCS (2 mA, 30 min) on sessions two and six with the anode placed over the left DLPFC. The authors concluded that tDCS therapy leads to improvements in working memory, and a positive effect on retention of learning [51]. Table 2 summarizes the principal studies above-mentioned. 

#### 2.1.3. Effect of tDCS on Attention Deficit/Hyperactivity Disorder (ADHD)

According to DSM-5, ADHD is a “persistent pattern of inattention and/or hyperactivity-impulsivity that interferes with functioning or development” with symptoms being present during childhood [20]. Similar to ASD, ADHD also has a very strong heritable component (~76%) and many genes have been associated with the disorder, some of them being also implicated in ASD [52]. Currently, the most common treatments for ADHD are pharmacological—stimulants and non-stimulants—and, though effective in reducing symptoms, either they do not seem to have a long-term effect, or induce relevant side-effects [53,54,55].

A deficit in the dopaminergic system seems to be at the root of ADHD. Dopaminergic neurons are directly activated by glutamate and inhibited by GABA. Researchers have suggested that a disequilibrium between GABAergic and glutamatergic neurotransmission may be present in the disorder [56,57,58]. Indeed, MRS studies showed altered levels of glutamate, glutamine [59] and GABA [60].

To investigate whether tDCS could impact on ADHD behavior and neurochemistry (Table 3), Leffa and colleagues [61] carried out a study using spontaneous hypertensive rat (SHR), a model for ADHD. The animals received anodal bicephalic tDCS over the PFC (0.5 mA) for 20 min over 8 consecutive days. Results showed that tDCS restores long-term memory deficits observed in SHR rats prior to the stimulation. An increase in the production of reactive oxygen species (ROSs) was also reported in the hippocampus and the authors postulate that it could be the result of increased dopaminergic neural activity. The increase in ROS was also accompanied by an increase in the antioxidant glutathione (GSH). However, the authors comment that this increase was not sufficient to prevent ROS production in SHR rats, requiring further studies on this issue [61].

TDCS has been used also in ADHD patients (Table 3). Indeed, fifteen teenagers (12–16 years old) with a diagnosis of ADHD (combined or hyperactive-impulsive type) received anodal tDCS (1 mA, 20 min) over the left DLPFC for five consecutive days. After the treatment, subjects showed a clear reduction of inattention, which became significant seven days after stimulation. There was also a reduction of hyperactivity after stimulation. Moreover, tDCS was well tolerated by the subjects, with no adverse effects reported [62]. Another study in adult ADHD patients (18–65 years old) with cortical anodal stimulation (2 mA, 20 min), concluded that after three sessions of tDCS subjects improved their performance on a measure of impulsivity [63]. Furthermore, in a study where sixteen patients diagnosed with ADHD (12–16 years old; combined or hyperactive-impulsive) received anodal tDCS applied over the left DLPFC for twenty minutes at an intensity of 1 mA, it was observed that stimulation led to larger activation of the left DLPFC and premotor cortex, as well as increased connectivity of the left DLPFC. These changes were associated with improved working memory performance, pointing to an association between increased connectivity caused by tDCS and increased interaction between DLPFC, working memory and executive function networks. The authors also reported that effects on functional connectivity persisted even twenty minutes after the end of stimulation, suggesting that these tDCS-related changes may cause improvements for ADHD [64]. Contrastingly, Westwood and colleagues [65] have reported no significant effects when anodal tDCS was applied over the right inferior frontal cortex (rIFC) and combined with cognitive training for fifteen weekly sessions, in a group of fifty boys (10–18 years) with ADHD, suggesting that tDCS over the rIFC does not have clinical benefits for the treatment of ADHD).

**Table 3:** Summary of studies on the effects of tDCS on Attention Deficit/Hyperactivity Disorder (ADHD).

Subjects	tDCS Intervention	Brain Region	Conclusion	Ref.
SHR rats	Anodal; 0.5 mA; 20 min; 8 consecutive days	Pre-frontal cortex	Restore long-term memory deficits, modulate neuroinflammatory molecules, and increase oxidative stress	[61]
Teenagers with a diagnosis of ADHD	Anodal; 1 mA; 20 min; 5 consecutive days	Dorsolateral prefrontal cortex	Reduction of inattention, and a reduction of hyperactivity	[62]
Adult ADHD patients	Anodal; 2 mA; 20 min	Dorsolateral pre-frontal cortex	Improve of performance on a measure of impulsivity	[63]
Teenagers with ADHD	Anodal; 1 mA; 20 min	Dorsolateral pre-frontal cortex	Improve of working memory performance.	[64]
ADHD patients (10–18 years old)	Anodal bifrontal; 1 mA; 20 min combined with cognitive training	Dorsolateral frontal cortex	No significant improvement of ADHD symptoms or cognitive performance	[65]

## 3. Conclusions and Future Perspectives

Multiple studies on the effects of tDCS in neurodevelopmental disorders have been carried out and more are underway. We have covered three main neurodevelopmental disorders where E/I balance is affected and can be potentially restored by tDCS: ASD, SCZ, and ADHD. TDCS can be used to modulate activity in neural circuits through its action on E/I balance and may be, therefore, a promising therapy for neurodevelopmental disorders. Clinical studies have shown encouraging results for the use of tDCS in ASD, SCZ, and ADHD to ameliorate symptoms. Reports are also consensual that tDCS is well tolerated by patients, even at a young age, with no serious adverse effects. This is a great advantage for neurodevelopmental disorders, as symptoms start at an early age, and often compliance to typical pharmacological treatments is difficult. Another advantage is that tDCS seems to work well as a stand-alone treatment, as well as in combination with other therapies, such as cognitive training, potentiating beneficial effects. Although it is still not well understood how tDCS and cognitive training improve cognitive function, its effects seem to be more effective for improving cognition, than cognitive training alone. Moreover, the protocols used until now were feasible and can be used in both children and adult population. When we compared tDCS “just stimulation” with tDCS with cognitive training, it seems that more functions were restored following the second option. More future studies will be needed to understand this issue.

Although animal studies have demonstrated the underlying mechanisms of tDCS’ effects on neural circuits, to this moment, the number of studies on animal models of neurodevelopmental, or neuropsychiatric, disorders are still surprisingly low. This may be due to the fact that tDCS has already been proved to be safe to use with humans, rendering human studies more appealing and popular. However, animal and in vitro studies may help shed a light on how tDCS works on different disorders, by allowing a more in-depth mechanistic analysis, combining non-invasive approaches with more invasive ones that would not be possible in human subjects. 

However, promising tDCS may seem as a possible therapy for neurodevelopmental disorders, these are still early stages and more research is necessary to better understand the effectiveness of tDCS, as well as its long-term effects. This will allow for treatment protocols to be optimized. It is, therefore, necessary to better understand how long the effects of tDCS last and how many consecutive sessions are more effective, so that dosage can be correctly administered in the clinical setting. Additionally, it would also be crucial to investigate how the positive effects of tDCS can be potentiated, and which patients would benefit more from the therapy. This would imply exploring the influence of physiological aspects, such as anatomy, age, sex, clinical history and severity of the disorder. It would also be worth further evaluating the importance of environmental factors, such as time of the day stimulation is administered, and montage factors, such as placement of electrodes and current intensity.

## Figures and Tables

**Table 1:** Summary of studies on the effects of tDCS on Autism Spectrum Disorder (ASD).

Subjects	tDCS Intervention	Brain Region	Conclusion	Ref.
Adults with high functioning ASD	Anodal; 2.0 mA; 30 min	Temporoparietal junction	Improvement of emotional processing	[32]
ASD children with Immature syntax	Anodal; 0.08 mA/cm^2^; 30 min	Dorsolateral prefrontal cortex	Improvement of syntax acquisition	[33]
ASD patients < 11 years of age	Cathodal; 1 mA; 20 min	Dorsolateral pre-frontal cortex	Significant decrease in the total score of three ASD clinical scales, accompanied by an improvement in autistic behavior up to six months after stimulation; increase in brain functional connectivity.	[34]
Male autism patients (5–8 years old) with mild to moderate autistic symptoms	Anodal; 1 mA; 20 min	Dorsolateral pre-frontal cortex	Improvements social/behavioral and health problems subscale	[35]
Adults with high functioning ASD	Anodal bifrontal; 1.5 mA; 40 min	Dorsolateral pre-frontal cortex	Improve working memoryperformance	[36]
ASD patients (10–18 years old)	Multi-channel anodal; 2 mA, 20 min	Temporoparietal junction	Ongoing	[37]
Male ASD patients (14–21 years old)	Cathodal and anodal; 1.5 mA, 20 min, 2 weeks with cognitive training	Left dorsolateral prefrontal cortex and and right supraorbital region	Promote social functioning	[38]

**Table 2:** Summary of studies on the effects of tDCS on Schizophrenia (SCZ).

Subjects	tDCS Intervention	Brain Region	Conclusion	Ref.
Adolescent MIS rats	Anodal or cathodal; 50 µA; 20 min × 2/day	Prefrontal cortex	Prevent positive neurological and behavior symptoms of schizophrenia	[48]
Patients with refractory auditory verbal hallucinations	Anodal and cathodal; 2 mA; 20 min × 2/day	Dorsolateral prefrontal cortex (anodal) and temporoparietal junction (cathodal)	Significant improvement on AHRS for up to 3 months.	[33,49]
SCZ patients18–25 years of age	Anodal and cathodal; 2 mA; 20 min × 2/day	Dorsolateral pre-frontal cortex (an-odal) and temporo-parietal junction (cathodal)	Amelioration of negative symptoms, except passive/apathetic withdrawal and stereotyped thinking, that lasted up to 6 weeks after the end of the trial.	[50]
SCZ patients	Eight cognitive training sessions (two session/day) combined with anodal, 2 mA, 30 min	Dorsolateral pre-frontal cortex	tDCS therapy leads to improvements in working memory, and a positive effect on retention of learning	[51]

MIS—maternal immune stimulation. AHRS—Auditory Hallucination Rating Scale.

## Data Availability

Not applicable.
